# Demonstration of the Use of Environmental DNA for the Non-Invasive Genotyping of a Bivalve Mollusk, the European Flat Oyster (*Ostrea edulis*)

**DOI:** 10.3389/fgene.2019.01159

**Published:** 2019-11-19

**Authors:** Luke E. Holman, Christopher M. Hollenbeck, Thomas J. Ashton, Ian A. Johnston

**Affiliations:** ^1^School of Ocean and Earth Science, National Oceanography Centre Southampton, University of Southampton, Southampton, United Kingdom; ^2^Xelect Ltd, Horizon House, Scotland, United Kingdom; ^3^Scottish Oceans Institute, School of Biology, University of St Andrews, Scotland, United Kingdom

**Keywords:** broodstock, hatchery management, single nucleotide polymorphism genotyping, mollusk aquaculture, minimally invasive sampling, non-invasive genetic sampling

## Abstract

Accurate SNP (single nucleotide polymorphism) genotype information is critical for a wide range of selective breeding applications in aquaculture, including parentage assignment, marker-assisted, and genomic selection. However, the sampling of tissue for genetic analysis can be invasive for juvenile animals or taxa where sampling tissue is difficult or may cause mortality (e.g. bivalve mollusks). Here, we demonstrate a novel, non-invasive technique for sampling DNA based on the collection of environmental DNA using European Flat Oysters (*Ostrea edulis)* as an example. The live animals are placed in individual containers until sufficient genetic material is released into the seawater which is then recovered by filtration. We compared the results of tissue and eDNA derived SNP genotype calls using a PCR based genotyping platform. We found that 100% accurate genotype calls from eDNA are possible, but depend on appropriate filtration and the dilution of the sample throughout the workflow. We also developed an additional low-cost DNA extraction technique which provided >99% correct SNP genotype calls in comparison to tissue. It was concluded that eDNA sampling can be used in hatchery and selective breeding programs applicable to any aquatic organism for which direct tissue sampling may result in animal welfare concerns or mortality.

## Introduction

Molluscan shellfish, such as clams, oysters, mussels, and scallops, represent around 20% of worldwide aquaculture production (FAO, 2019). Although the life cycle has been closed for many mollusks, most aquaculture production is still dependent on unpredictable collection of spat (settled larvae) from the wild. The availability of wild spat can be negatively impacted by overfishing, environmental or trophic changes (Waldbusser et al., 2015; Lagarde et al., 2018), and disease outbreaks (Boudry et al., 1998; Garcia et al., 2011; Murray et al., 2012), none of which are under the control of the producer. There is therefore a trend towards hatchery-based production of juveniles for on-growing in the sea. Hatchery-based production allows for genetic improvement of stock *via* selective breeding, which has the potential to improve economically important traits such as growth and disease resistance by 10%–15% per generation (Hollenbeck and Johnston, 2018).

One particular challenge of molluscan aquaculture is the availability of non-invasive DNA sampling techniques for parentage assignment and advanced marker-assisted or genomic selection strategies. Current DNA collection strategies for mollusks involve the use of anesthetic chemicals to relax internal muscles which opens the shell to enable clipping of internal tissues such as the gill or mantle which are not accessible when the shell is closed (Suquet et al., 2009; Suquet et al., 2010; Mao et al., 2013). An alternative method is to sample internal fluids using a syringe with or without anesthetic (Jones et al., 1993; Kurita and Kijima, 2019). These methods can result in a physiological stress response (Butt et al., 2008; Granados-Amores et al., 2017) and in some cases cause mortality of valuable broodstock (Henley et al., 2006). Furthermore, clipping of internal tissues such as mantle tissue which is rich in mechanoreceptors and chemoreceptors is problematic from an animal welfare perspective, particularly for small species or for individuals that are immunocompromised or in poor condition.

Recent advances in the isolation of environmental DNA (eDNA) potentially offer a non-invasive alternative to tissue sampling (Carroll et al., 2018). eDNA is a polydisperse mixture of nucleic acid containing material shed from an organism and isolated from environmental samples such as sediment or water (Thomsen and Willerslev, 2015; Deiner et al., 2017). The majority of eDNA studies have been used to test ecological hypotheses either by recording the incidence of a single aquatic species using species-specific primers (Collins et al., 2018; Seymour et al., 2018) or many species simultaneously with metabarcoding (Stat et al., 2017; Deiner et al., 2018; Holman et al., 2019). Overall, eDNA has been shown to be highly accurate and at least as sensitive as other biodiversity monitoring techniques (Deiner et al., 2017). Additionally, studies have shown that eDNA can provide population genetic inference both in the laboratory (Espinoza et al., 2017) and in coastal ecosystems (Sigsgaard et al., 2017; Stat et al., 2017). In aquaculture species, eDNA has recently been used for the detection of bacterial and parasitic diseases (Nguyen et al., 2018; Peters et al., 2018).

The aim of the present study was to determine whether eDNA could be used to genotype individual bivalves at multiple SNP loci with the accuracy required for parentage assignment. We tested both low cost and archive grade eDNA extraction methods and developed a protocol that achieved 100% accurate genotype calls in comparison to tissue samples from the same individuals. The use of eDNA for the non-invasive genotyping of bivalve broodstock and their offspring represents an important new tool for the development of hatchery-based selective breeding programs.

## Materials and Methods

### Animals and Water Sampling

Six European flat oysters (*Ostrea edulis* L.), 30–80 g, were acclimatized in a 50 L seawater aquarium at 16°C for 60 days, with 700 L/hour external filtration (Ehiem, Deizisau, Germany). Twenty percent of aquarium water was replaced weekly with fresh sea water. The oysters were from aquaculture populations obtained from Loch Nell Oysters (Argyll, UK) and are derived from native stock from the Argyll area. During acclimation, the oysters were fed a maintenance diet of powdered algal biomass (Megatech Research, Switzerland). Each oyster was externally rinsed with reverse osmosis (RO) filtered water, then placed into a polypropylene vessel with 500 ml seawater made from artificial salt (Red Sea Aquatics Ltd, London, UK) dissolved in (RO) water to 33ppt salinity. Duplicate water samples of 75 ml were taken from each vessel 72 h after the oyster was introduced. The oysters were sacrificed and a 5 mm^2^ section of mantle was dissected and stored in 100% ethanol until DNA extraction. A 75 ml artificial seawater control sample was taken before filling the vessels. All 75 ml water samples (experimental and control) were filtered using a vacuum filtration manifold and 47 mm 0.45 µm Cellulose Nitrate filters (Sartorius, Göttingen, Germany) in a glass housing.

All reused equipment was soaked in 0.5% sodium hypochlorite solution (household bleach solution diluted 1 in 10 with RO water) for 1 hour before the start of the experiment. Filtration equipment was thoroughly washed between sampling and 100 ml bleach solution (as above) followed by 200 ml RO water was filtered between every sample to minimize the possibility of cross contamination.

### DNA Extraction

Approximately 25 mg mantle tissue was dissected and finely sliced with a sterile scalpel. DNA was extracted using the DNeasy Blood and Tissue Kit (Qiagen, Hilden, Germany) under the manufacturer’s recommended protocol with a final DNA elution in 100 µl of PCR cert. water. All eDNA filters were sliced into ∼3 mm sections using a sterile scalpel. One replicate at each sample point was subject to DNA extraction using the DNeasy Blood and Tissue kit. Briefly, 80 µl of Proteinase K solution (20 mg/ml) and 720 µl of Qiagen ATL Buffer was added to each sliced filter and thoroughly vortexed followed by overnight digestion at 56°C. Five hundred microliters of lysate was mixed with 500 µl of Buffer AL and 500 µl of 100% ethanol, DNA extraction proceeded as in the manufacturer’s protocol, with the entire 1,500 µl of lysate being passed through the extraction column. DNA was eluted in 60 µl of PCR grade water. A second eDNA replicate was subject to a crude, low-cost DNA extraction (Walsh et al., 1991) in which 800 µl of 10% Chelex 100 (Sigma-Aldrich, St Louis, USA) suspension containing 0.2 mg/ml Proteinase K was added to the filter. The mixture was then thoroughly vortexed and incubated at 56°C for 60 minutes, 60 µl of lysate was removed and incubated at 95°C for 10 min and then stored at -20°C. DNA concentration was calculated using the Quant-iT PicoGreen dsDNA Assay Kit (ThermoFisher Scientific, Waltham, USA) on the Agilent (Santa Clara, USA) Mx3000P qPCR instrument using the protocol described in Blotta et al. (2005).

### Genotyping

**Figure 1:**
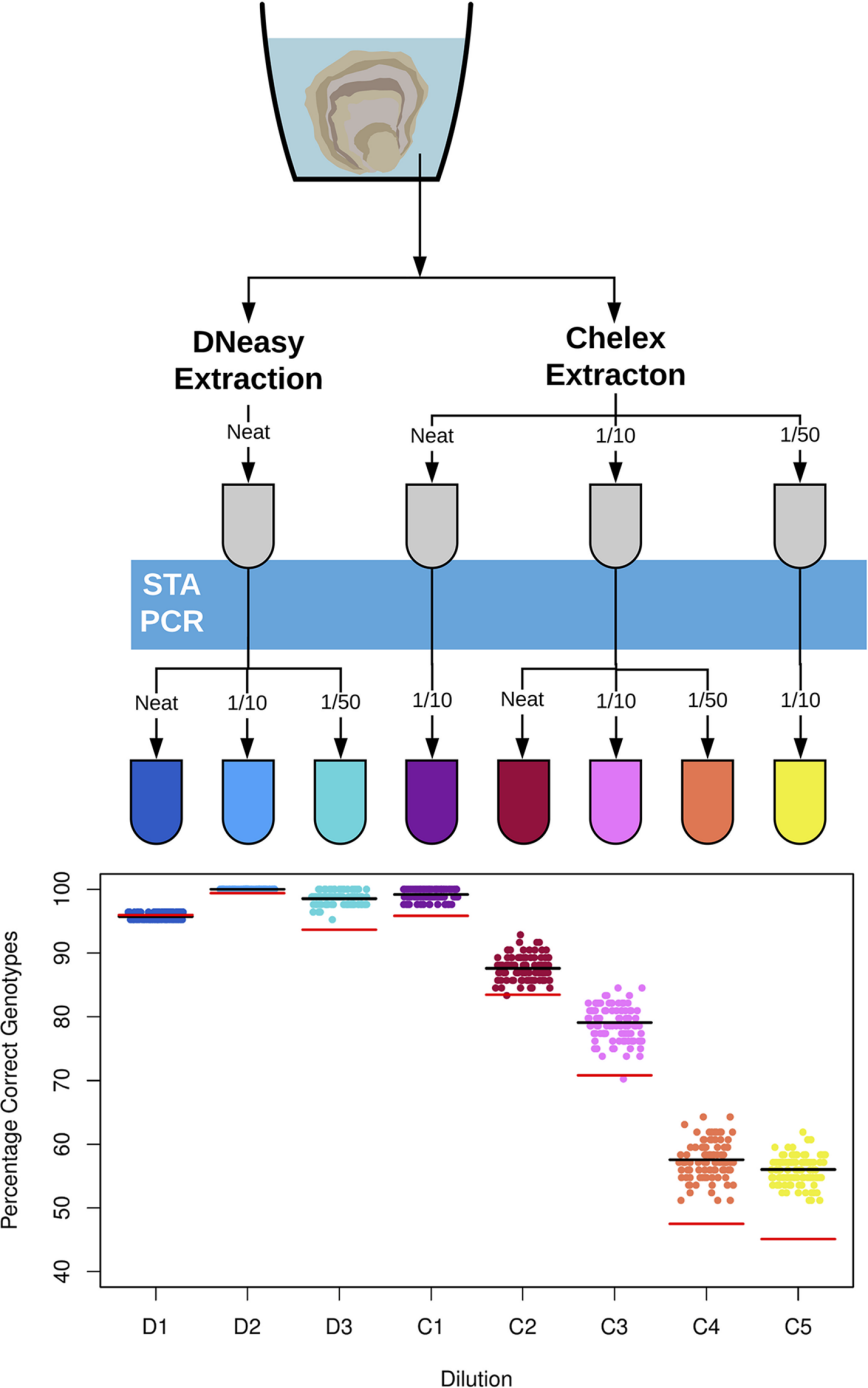
Diagram (top) detailing dilutions of DNA template from different DNA extraction techniques used for SNP genotyping of environmental DNA for *Ostrea edulis*. The two DNA extraction methods, the Qiagen DNeasy Blood and Tissue Kit and the crude Chelex extraction, are shown at the top. DNA samples are subject to dilution step before and after the Specific Target Amplification PCR (STA PCR), here shown as a blue bar. All dilutions were with PCR grade water and samples that were not diluted are labelled ‘neat’. A scatter plot (bottom) shows the percentage of correct environmental DNA derived genotype calls in comparison to the tissue extractions for the eight dilutions. Each point represents the total result derived from a random sample of three replicates per individual oyster genotype. The black line indicates the average percentage correct eDNA genotypes across the 100 random samples. The red line indicates the average percentage correct eDNA genotypes across the 100 samples if only a single replicate is used for each individual oyster genotype.

*O. edulis* SNP sequence data from Gutierrez et al. (2017) was sorted by mean minor allele frequency (MAF) across the discovery populations. SNPs used in parentage assignment are frequently selected for high MAF i.e. > 0.2 for better discrimination between individuals (Holman et al., 2017). This property also makes them appropriate for checking genotype accuracy. Therefore the 16 SNPs with the highest mean MAF were sent to the Fluidigm D3 assay design portal for synthesis of Fluidigm SNP Type genotyping assays (**Supplementary Table 1**). Genotyping proceeded with the Fluidigm EP1 platform using the manufacturer’s protocols (Fluidigm Ltd., San Francisco, USA). Briefly, template was subject to a multiplex PCR containing primers for all 16 target regions. This Specific Target Amplification step (STA) increases target copy number, making the template amenable to amplification in microfluidic chambers. The STA product was then loaded on to a Fluidigm 96.96 Dynamic Array genotyping chip along with assays in sextuplicate, followed by a PCR at manufacturer recommended conditions and imaging on the Fluidigm EP1 data collection system. The quantity of target DNA in environmental DNA is highly dilute compared to DNA template samples typically used on genotyping platforms (e.g. tissue, cell culture). Therefore, sample dilutions recommended in the manufacturer’s protocol were predicted to dramatically alter the accuracy of genotype calls. Dilution of DNA samples before STA is important to minimize the amount of potentially PCR inhibiting co-purified contaminants from DNA extractions, while also transferring an appropriate amount of DNA template for the PCR reaction. Dilution after the STA is a balance between diluting unused reagents from the PCR while transferring enough target copies for successful fluorescence-based genotyping. With these limitations in mind, several different dilutions with RO water were trialled as shown in **Figure 1**.

### SNP Genotyping Analysis

SNP genotypes were called using k-means clustering under the default settings in the Fluidigm Genotyping Analysis Software. Calls were checked manually to ensure clustering was performed appropriately within dilutions. Genotypes for eDNA samples were called in relation to the tissue samples from the equivalent dilution.

To evaluate eDNA genotyping success, four metrics were evaluated. Overall SNP call rate was calculated as the proportion of allele calls across the 96 assays (16 SNPs, 6 replicates per SNP) for the eDNA sample that matched the tissue sample. Replicate SNP reactions were considered independent and “no calls” were included in calculations. This metric evaluated both the overall genotyping success of the Fluidigm EP1 system and also the congruence between results from eDNA and tissue DNA samples.

SNP genotype success was calculated by randomly sampling data points from the six replicates to simulate different levels of replication. Scenarios with three, two and a single replicate were simulated one hundred times. In each simulation the majority genotype, excluding “no calls”, from the subsample was compared to the tissue derived genotype. In the case when there were two or more conflicting genotypes of equal frequency in a simulation, the call was marked incorrect.

## Results

No mortality was recorded and oysters showed no visible sign of spawning before, during, or after eDNA sampling. DNA was successfully extracted from all samples. Oyster tissue samples assayed with Picogreen contained 45.9 ± 21.3 (s.d.) ng/µl of dsDNA, control samples contained 0.48 ± 0.47 ng/µl of dsDNA, and eDNA samples from the DNeasy extraction contained 115.4 ± 50.6 ng/µl of dsDNA.

Out of the 16 trialed assays two failed to produce any identifiable clusters, indicating no polymorphism among the tested oysters or a non-functioning assay. In all cases the tissue samples for the remaining 14 assays gave high quality clusters reliably identified using k-means clustering (SNP call data provided in **Supplementary Table 2**).

Across all time points and samples, the average correct genotyping reaction call rate for eDNA samples compared to the tissue samples was 78.1 ± 24.8% (s.d.) with a maximum correct call rate of 100.0% and a minimum correct call rate of 19.0% (**Supplementary Figure 1**).

Across the 100 simulated genotyping scenarios, dilutions D2 and C1 provided the highest accuracy between eDNA and tissue DNA genotypes for the DNeasy and Chelex extractions, respectively, as shown in **Figure 1**. For the D2 dilution, all 100 simulated scenarios under duplicate and triplicate replication gave 100% correct genotypes, with an average of 99.4% accuracy across the 100 scenarios with a single replicate (see **Supplementary Figure 2** for duplicate simulation results). The C1 dilution gave a mean accuracy of 95.8%, 98.5%, and 99.2% across the 100 scenarios with one, two, and three replicates, respectively. As shown in **Figure 1**, all other Chelex dilutions provided poor accuracy across the scenarios with mean values of less than 90% in all cases.

## Discussion

Here we show that the collection of eDNA can be used to accurately genotype bivalve mollusks and potentially other aquatic organisms. The influence of DNA extract dilution on genotyping accuracy was assessed to produce a practical protocol for the European flat oyster that can be used by researchers and aquaculture professionals as a template to develop viable alternatives to invasive tissue sampling in similar species. We also demonstrated that eDNA extracted using this protocol is of sufficient quality and quantity for multi-locus genotyping, which is necessary for most applications in aquaculture breeding programs.

The protection offered by an external shell structure provides an evolutionary advantage for many invertebrate taxa. However, in the context of selective breeding programs this is a disadvantage, increasing the force or invasiveness required to sample DNA compared to organisms with no shell. Invasive methods involving the removal of internal tissue or fluid are routinely reported in marine and freshwater mussels (Yanick and Heath, 2000; Gustafson et al., 2005; Henley et al., 2006), oysters (Culloty and Mulcahy, 1992; Suquet et al., 2009; Suquet et al., 2010; Lokmer and Wegner, 2015), and scallops (Mao et al., 2013). Although, methods have been developed for minimally invasive DNA sampling of finfish using external mucus (Le Vin et al., 2011; Taslima et al., 2016), non-invasive methods are lacking for mollusks. The method presented here involves no contact beyond the handling required to place and remove the individual from the sterile seawater chamber, potentially saving on labor costs as well as achieving a higher animal welfare standard. The equipment required is readily available, relatively inexpensive and provides high quality DNA for downstream applications.

The eDNA genotyping method presented here was developed using a species of comparatively little aquaculture interest. A question that remains is to what extent this method is applicable to other mollusks and aquaculture species. Previous studies have documented that species relevant to aquaculture such as salmonid fish (Atkinson et al., 2018), oysters (Holman et al., 2019), and scallops (Bayer et al., 2019) all produce a sufficient quantity of eDNA for sensitive detection in ecological experiments. Difficulty has only been documented in isolating sufficient eDNA in an invasive crab species (Forsström and Vasemägi, 2016). We can therefore expect almost all aquaculture species to produce eDNA of sufficient yield for the method presented here to provide a non-invasive DNA sample for downstream genetic inference.

Ecological studies have shown a dramatic effect of DNA extraction technique on the results of both eDNA metabarcoding (Djurhuus et al., 2017; Deiner et al., 2018) and qPCR experiments (Hinlo et al., 2017). Our data corroborate these findings, showing that two different eDNA extraction techniques provide variable success in fluorescence-based genotyping, and that a column-based extraction provides greater average genotype accuracy in comparison to a crude lysis technique, albeit at higher cost. We additionally found that dilution of pre- and post-PCR products had an effect on correct genotype calls, decreasing the mean accuracy of genotype calls by over 40% in Chelex extractions. Studies have shown that dilution of eDNA samples has a negative effect on species detectability (McKee et al., 2015; Piggott, 2016), but little work has explored how sample dilution affects SNP genotype accuracy. These results therefore indicate that eDNA users should be wary of diluting samples for accurate genotyping of SNPs or in the estimation of haplotype frequencies.

Small panels of 100–500 SNP markers are the genetic markers of choice for parentage assignment and the determination of relatedness in modern aquaculture breeding programs, allowing both the estimation of breeding values and the control of inbreeding (Robledo et al., 2018). Large panels of SNPs (1,000–50,000) have been used in aquaculture for generating linkage maps (Li and He, 2014), estimating trait heritability (Gutierrez et al., 2018), quantitative trait locus (QTL) mapping (Jiao et al., 2014), genomic selection (Palaiokostas et al., 2016), and most recently the estimation of effective population size (N_e_) (D’Ambrosio et al., 2019). SNP arrays (a florescence-based DNA microarray SNP genotyping method) are commonplace for genotyping thousands of SNPs in advanced aquaculture breeding programs. Further work should investigate if the proportion of total isolated eDNA corresponding with the target organism (and not associated bacteria) is of sufficient quality and quantity for other applications than parentage assignment.

Overall, the use of eDNA for SNP genotyping described here will facilitate broodstock management and animal welfare in delicate or hard to sample animals which are enclosed in an external shell or exoskeleton by reducing handling stress and associated mortality.

## Data Availability Statement

Raw Illumina sequencing data used to identify variants in Gutierrez et al. (2017) that are genotyped in this work is available at the European nucleotide archive under accession number PRJEB20253 (http://www.ebi.ac.uk/ena/data/view/PRJEB20253). All other datasets used in the study are contained in the article/**Supplementary Material**.

## Author Contributions

LH, TA, and IJ contributed to the conception and design of the study. LH performed the laboratory work, analyzed the data, and produced the figures. LH and CH wrote an initial draft manuscript. All authors contributed to manuscript revision, read, and approved the final version.

## Funding

This project has received funding from the European Marine Biological Research Infrastructure Cluster (EMBRIC) project funded by the European Union’s Horizon 2020 research and innovation program under grant agreement No 654008. LH was supported by the Natural Environmental Research Council (grant number NE/L002531/1).

## Conflict of Interest

LH is supported through a CASE studentship by Xelect Ltd. IJ, TA, and CH are employees of Xelect Ltd. The remaining authors declare that the research was conducted in the absence of any commercial or financial relationships that could be construed as a potential conflict of interest.
