# Supplementary material for: Demonstration of the Use of Environmental DNA for the Non-Invasive Genotyping of a Bivalve Mollusk, the European Flat Oyster (Ostrea edulis)
Source: Front Genet. 2019 Nov 19;10:1159. doi: 10.3389/fgene.2019.01159 (PMC6877716; doi:10.3389/fgene.2019.01159)
Supplement: Supplementary file 1 [file Presentation_1.pdf]

1.1 Supplementary Figures

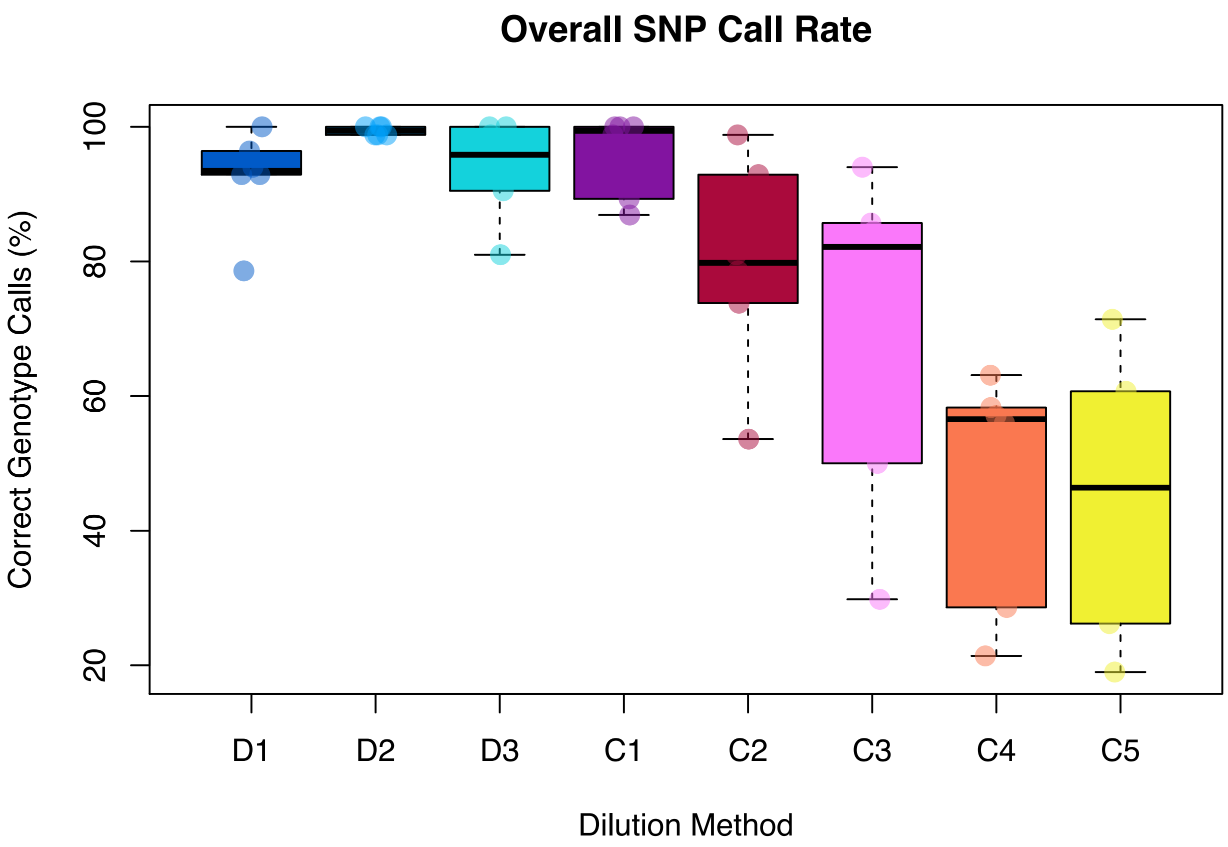

Supplementary Figure 1. Boxplot detailing overall proportion of eDNA derived SNP genotype calls compared to tissue derived calls for each *Ostrea edulis* individual. Each colored box shows results for the dilutions as detailed in the main manuscript. The central black of each box is the median value, the box edges are the 25th and 75th percentiles and the outlier whiskers indicate the nearest value within 1.5 times the interquartile range. Raw data points have been overlaid on each box.

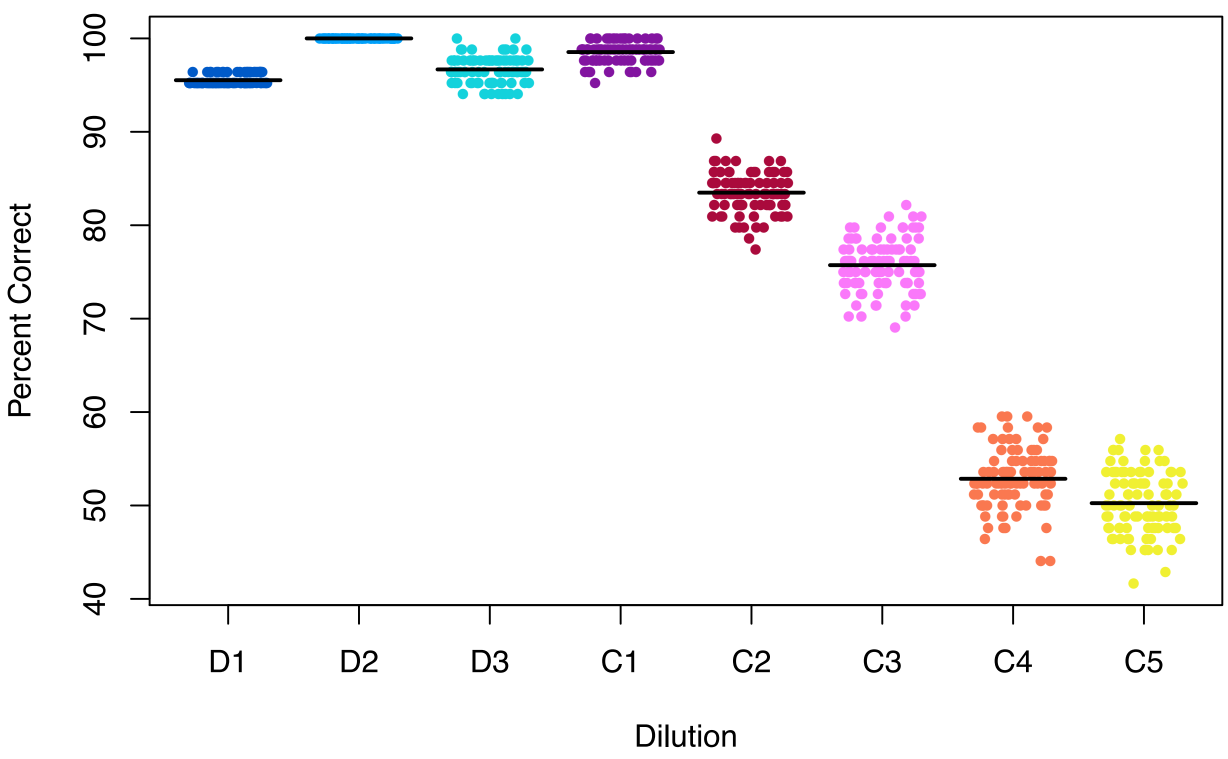

Supplementary Figure 2. Scatterplot detailing percentage of correct environmental DNA derived genotype calls in comparison to the tissue extractions for the eight dilutions detailed in the main manuscript. Each point represents the total result derived from a random sample of two replicates per individual *Ostrea edulis* genotype. The black line indicates the average percentage correct eDNA genotypes across the 100 random samples

Supplementary Tables

Supplementary Table 1. Table detailing SNPs used in the present study, sequence information and minor allele frequency in genotyped animals.

Supplementary Table 2. Table detailing SNP genotype calls for both eDNA and tissue samples.
